# Gamification of graduate medical education in an emergency medicine residency program

**DOI:** 10.1186/s12245-022-00445-1

**Published:** 2022-08-30

**Authors:** Shayne Gue, Joseph Ray, Latha Ganti

**Affiliations:** 1 Emergency Medicine Residency Program, AdventHealth Orlando, Orlando, FL USA; 2 Emergency Medicine Residency Program, University of Central Florida/HCA Healthcare GME (Greater Orlando), FL Orlando, USA

**Keywords:** Gamification, Serious games, Medical education, Adult learning, Team-based learning

## Abstract

**Objectives:**

Our program implemented East EMWars, a year-long, longitudinal game that added competition to our existing curricular content. We surveyed residents to investigate the impact of gamification in emergency medicine residency training. We hypothesized that residents would report higher levels of motivation, engagement, and challenge with gamification compared to traditional didactics. Furthermore, we hypothesized that residents would exhibit generally positive perceptions about gamification as a learning tool and that it would translate to improved performance on the annual in-training examination.

**Methods:**

This was a single-center, prospective pre- and post-intervention survey study at a community-based emergency medicine residency program. Given the multiplicity of research questions and inherent nature of educational research, a mixed methods approach was utilized. We utilized nonparametric testing for quantitative data with paired responses pre- and post-intervention. We solicited comments on the post-intervention that were categorized under thematic approach and presented in complete and unedited form in the results.

**Results:**

Eighteen (100%) of eligible residents in our program participated in both surveys. There were statistically significant increases in reported levels of motivation, engagement, and challenge with gamification compared to traditional didactic methods. Residents also reported overwhelmingly positive general perceptions about gamification and its broader generalizability and applicability. We did not reach statistical significance in determining if in-training exam scores were associated with our gamification initiative.

**Conclusions:**

This study was a first-of-its-kind look into a longitudinal game in an emergency medicine residency program. Although our results are encouraging, medical educators need further research to determine if this increase in motivation, engagement, and challenge will be associated with an increase in examination scores or, more importantly, healthcare outcomes. Theory-based, broader-scale, prospective studies are needed to further explore and help establish these associations and outcomes.

**Supplementary Information:**

The online version contains supplementary material available at 10.1186/s12245-022-00445-1.

## Introduction

What really is gamification? Ten years ago, it was unheard of. Now, it is in the armory of many educators across the country. Gamification has been defined as “the use of game design elements in non-game contexts” [1] or “the craft of deriving all the fun and addicting elements found in games and applying them to real-world or productive activities.” [2] But how do we create it, apply it, and explore the potential benefits to resident education?

Long before we had the terminology, gamification existed in popular culture. The American Airlines’ frequent flyer program began in 1981, rewarding loyal customers with incentives as they ascended the ranks of the program. More recently, gamification has made its way into most other areas of daily life; Starbucks is one example which gives users a platform to earn rewards and a mobile payment method all in one [3]. Despite a more recent uptick in research on gamification in the educational realm, there remains very little specific to graduate medical education [4–6].

Although emergency medicine may be leading the way, existing gamification projects are typically single sessions, narrowly focused on a particular content area, and heavily reliant on quiz-style gaming [7–15]. Instead, we sought to investigate the impact of a long-running game held longitudinally throughout an entire academic year. This innovation allowed for team building and competition over the course of an entire academic year, rather than a single session, or shorter term. While there were no changes made to the methods for curricular content delivery, we sought to investigate the impact of adding gamification elements to existing curricula to determine levels of motivation, engagement, and challenge compared to non-gamified traditional didactic sessions. We hypothesized that residents would report increased levels in all categories participating in the gamified model compared to the traditional didactic curriculum. We hope to encourage future educational researchers to design studies that will help to determine if this increased motivation, engagement, and challenge may also lead to improved knowledge translation, better performance on certifying examinations, and improved outcomes in patient care.

## Methods

### Study design and population

This was a prospective, observational research study designed to explore the perspective of emergency medicine residents in a newly implemented gamified graduate medical education curriculum. Given the multiplicity of research questions and inherent nature of educational research, a mixed methods approach was utilized. Surveys were administered to participants before and after their participation in a longitudinal game, held over a 10-month period between August 2020 and June 2021. The study was approved under exempt review by the AdventHealth Orlando Institutional Review Board.

Our study took place in a single-center, community-based, 3-year emergency medicine program in the southeast. Participants were voluntarily enrolled from the pool of 18 emergency medicine residents currently in the program. Participation was 100%. Several measures were taken to avoid any type of coercion to participate in the study.

Of note, the implementation of the game was an innovation to our existing curriculum, and as such, participation was required as a normal part of conference attendance and progression through our training program. Participation in this research study, by completing pre- and post-intervention surveys, was completely voluntary, and residents received no incentive to participate or consequence to not participate. For more information about game design, play mechanics, and scoring, see attached (Additional files 1 and 2: Appendices A and B).

### Survey content and administration

Participants were recruited from the available pool of residents in our program. A flyer advertising the study was placed in the resident sign-out room within the emergency department. Additionally, after an overview presentation about the game, which was given during regularly scheduled didactic conferences, the investigators provided detailed verbal information about the game and study (completing the surveys), held a Q and A session, and presented the letter of invitation to those residents that expressed interest in participating.

Two surveys were distributed to all participants. The pre-intervention survey was distributed prior to the initiation of the game (August 2020) and included questions regarding perceived level of motivation, engagement, and challenge with the current (non-gamified) didactic curriculum as well as their perceptions about the implementation of gamification. The post-intervention survey was distributed after the completion of the game (June 2021) and included questions regarding perceived level of motivation, engagement, and challenge with the elements of gamification that had been added to the curriculum in comparison with the traditional didactic curriculum. This survey also solicited their perceptions about feasibility and generalization of gamification in graduate medical education.

Paper surveys were distributed in sealed envelopes with each participant’s name printed on the front. Each survey was uniquely coded so that responses remained anonymous but were matched pre-intervention and post-intervention to the same participant for data analysis purposes. All 36 surveys were printed and sealed in envelopes prior to starting the study. This ensured we were able to pair responses between survey periods but maintain anonymity of each individual resident’s responses. Surveys are attached (Additional files 3 and 4: Appendices C and D).

### Data analysis

The primary objective of this study was to evaluate resident motivation, engagement, and challenge with a longitudinal, gamified medical education curriculum. The secondary objective was to evaluate whether increased participation with this gamified curriculum translated to improved scores on the annual in-training examination. Levels of motivation, engagement, and challenge were analyzed with the Wilcoxon signed-rank test, a nonparametric test comparing pre- (traditional didactic curriculum) and post-intervention (gamified curriculum) survey responses on a 5-point Likert scale. In-training examination percentile scores were self-reported via survey, where second- and third-year residents reported their 2020 ITE percentile score on the pre-intervention survey, while all respondents were asked to report their 2021 ITE percentile score on the post-intervention survey. Open-ended survey questions were summarized using qualitative methods, specifically thematic approach. Given this triangulation model of mixed methods data collection, there existed a possibility for contradiction in quantitative versus qualitative data [16]. Educational researchers have suggested that if resource limitations prevent further exploration of these contradictions, the results should be presented together, and authors should define directions for future studies [17].

## Results

Of the 18 eligible participants, all 18 (100%) completed all required items on both pre-intervention and post-intervention surveys. Among the participants were 6 (33.3%) first-year residents, 6 (33.3%) second-year residents, and 6 (33.3%) third-year residents. The primary objective of the study was to evaluate resident motivation, engagement, and challenge with a longitudinal, gamified medical education curriculum. The secondary objective was to evaluate whether increased participation with this gamified curriculum translated to improved scores on the annual in-training examination.

### Motivation, engagement, and challenge

For motivation, 13 (72.2%) respondents indicated increased motivation with a gamified curriculum compared to traditional didactics as evidenced by higher scores on a 5-point Likert scale post-intervention paired to pre-intervention responses; 3 (16.7%) reported no difference, and 2 (11.1%) had decreased motivation scores. The Wilcoxon signed-rank test supported rejection of the null hypothesis (*p*-value = 0.0028), so we concluded there is a significant increase in resident motivation.

For engagement, 13 (72.2%) respondents indicated increased engagement with a gamified curriculum compared to traditional didactics as evidenced by higher scores on a 5-point Likert scale post-intervention paired to pre-intervention responses; 5 (27.8%) reported no difference, and none had decreased engagement scores. The Wilcoxon signed-rank test supported rejection of the null hypothesis (*p*-value = 0.0002), so we concluded there is a significant increase in resident engagement.

For challenge, 10 (55.6%) respondents indicated increased challenge with a gamified curriculum compared to traditional didactics as evidenced by higher scores on a 5-point Likert scale post-intervention paired to pre-intervention responses, 7 (38.9%) reported no difference, and 1 (5.6%) had decreased challenge scores. The Wilcoxon signed-rank test supported rejection of the null hypothesis (*p*-value = 0.0068), so we again concluded there is a significant increase in resident challenge. Descriptive statistics are displayed in Table 1.

**Table 1:** Descriptive statistics for motivation, engagement, and challenge

	Motivation		Engagement		Challenge	
	Pre	Post	Pre	Post	Pre	Post
Mean	3.3	4.3	3.4	4.6	3.5	4.4
Median	3	4.5	4	5	4	4
Mode	3	5	4	5	4	4
IQR	1 (3, 4)	1 (4, 5)	1 (3, 4)	1 (4, 5)	1 (3, 4)	1 (4, 5)

1 strongly disagree, 2 disagree, 3 neutral, 4 agree, 5 strongly agree

### In-training exam scores

In-training exam percentile scores were self-reported by second- and third-year residents on both pre- and post-intervention surveys. Scores were reported in ranges of 10 percentile points and coded to a 10-point scale (1st–9th percentile = 1, 10th–19th percentile = 2, 90th–99th percentile =10, etc.). There were a total of 12 respondents, 6 (50%) second-year residents and 6 (50%) third-year residents. Five (41.2%) respondents indicated an increase in ITE percentile scores after intervention, 2 (16.7%) respondents had no change in ITE percentile scores, and 5 (41.2%) respondents indicated an decrease in ITE percentile scores. A paired *t*-test was conducted (*p*-value = 0.198), failing to reject the null hypothesis. Subsequent power analysis (0.64) determined that the study was insufficiently powered to detect a difference if one exists. Unfortunately, we were limited by the number of eligible participants, not to mention a myriad of confounding factors that limit the usability of the obtained data.

### General perceptions of gamification

Lastly, we asked participants several questions regarding their perceptions of gamification on the post-intervention survey. Respondents were asked to rate their level of agreement with several statements on a 1 to 5 Likert scale, from “strongly disagree” to “strongly agree.” Results are summarized in Table 2. Overwhelmingly, residents indicated agreement with positively worded statements about the impact of gamification on their educational experience.

**Table 2:** Descriptive statistics for perceptions on gamification

	Competition drives performance in a gamified curriculum model	I expanded my knowledge as a result of the gamified curriculum model	Gamification should be continued and increased in the future	I prefer to work in groups rather than solo when learning new materials	I prefer interactive learning (e.g., questions or games) rather than traditional methods (e.g., reading text or watching videos/lectures)
Mean	4.6	4.2	4.7	4.1	4.4
Median	5	4	5	4	5
Mode	5	4	5	5	5
IQR	1 (4, 5)	1 (4, 5)	0.75 (4.25, 5)	2 (3.5)	1 (4, 5)

1 strongly disagree, 2 disagree, 3 neutral, 4 agree, 5 strongly agree

Residents also provided summative qualitative feedback through open-ended comments on the post-intervention survey. Their complete responses are included in Table 3 below. All comments were rated positively and associated with positive perceptions of gamification. Comments were categorized into “general” and categories specific to certain aspects of our game.

**Table 3:** Qualitative feedback through anonymous comments

*Item no. 13 on post-intervention survey solicited “comments” (open-ended)*	
Theme: general comments	
“Well thought out” “Loved it” “It was great playing EMWars as a resident” “Loved the competition” “Great project! Keep it going!” “Gamification is the best thing to happen to our curriculum during my 3 years in residency” “Gamification was a great way to learn and reinforce learning” “I feel that over the past year, new sessions were more engaging and easier to remember”	
Theme: procedures	
“It was enjoyable and helped improve procedures documented” “Encouraged me to log in more procedures even if I had reached all required to graduate”	
Theme: team building	
“The team build a lot of cohesiveness by keeping each other accountable”	

## Discussion

In this prospective, pre- and post-intervention survey, we determined that residents in an emergency medicine program perceive increased engagement, motivation, and challenge in a gamified curricular model compared to traditional, non-gamified, lecture-based didactics. Residents reported overwhelming agreement with positive statements regarding the implementation of gamification in their educational experiences, but our study was not powered to detect any significant difference in in-training examination scores before and after the intervention. Furthermore, there are a multitude of confounding variables affecting examination scores that were unable to be controlled for in this specific study.

For over half a century, educational researchers have recognized the need for change in our approach to adult learning [18]. Traditional pedagogical methods have long been shown to fall short. Couple this with an upcoming generation of adult learners who grew up in the technological age, not knowing life (or education) without computers, cellphones, and the internet. Emergency medicine educators have been leading the way in innovative strategies to improve adult learning for this new generation. Gamification is just one approach.

Over the past decade, the implementation of gamification models into educational curriculum has been met with tremendous success in a variety of settings [4, 5]. Systematic reviews of gamification in health professions education confirm that gamification has attracted the attention of educational researchers during the last few years, but there remains a scarcity of evidence and need for further theory-driven research [19–21]. In emergency medicine, we have seen success in gamification in various forms: game show-style quizzing [7–9], simulation competitions [10–12], escape rooms [13–15], and more. However, to our knowledge, this is the first study to consider these impacts of gamification in a longitudinal fashion.

The results supported our primary hypothesis that the game would increase learner motivation, engagement, and challenge. Selecting motivation as a measurable variable was important in that we believe it is a necessary precursor to self-directed learning. And the motivation we incur from gamification is the likely reason for its perceived success.

Perhaps the most broadly researched macro-theory of motivation is self-determination theory. SDT describes the core incentives of human behavior to reside with the needs for competence, relatedness, and autonomy [22]. Making learning into a game can allow residents the autonomy to set goals and obtain achievements, relate and socialize with their colleagues, and show their competence with progression in the game. In addition to these intrinsic needs, our game added extrinsic motivators in the form of gift cards and other prizes as awards for achievement. This was a strategic addition to solicit buy-in and further increase attention, engagement, and motivation. Although extrinsic motivators, like rewards and prizes, must be utilized cautiously, they have the ability to increase engagement, which can assist learners in identifying the value of an educational activity as it may apply to their circumstances, leading to autonomous motivation or “motivation arising out of genuine interest or personal endorsement or valuing of an activity.” [23]

Although our study was not powered to detect a significant difference in in-training examination scores, SDT supports that increasing levels of motivation, engagement, and challenge will lead to improved learning and outcomes. Further prospective, theory-based research is needed to determine whether gamification in emergency medicine residency programs can lead to improvements in both board examination scores and pass rates as well as patient outcomes.

### Limitations

Medical education research, in general, suffers many methodological limitations [24]. Our study was no exception. This study was conducted at a single, community-based residency program limited to a very small sample size of only 18 residents, even with 100% participation. Furthermore, survey data, especially data obtained from residents, is prone to observer bias and social desirability bias as residents know they are being studied and may select the responses they believe to be desired by the investigators [25, 26].

Our main limitation, however, was the lack of power to detect a significant difference in performance on the in-training exam, likely due to small sample size. Even still, there are multiple confounding variables that affect examination scores and would prove difficult to control across a 1-year period separating subsequent in-training exams: other curricular components, quality of conferences, clinical experiences, self-directed learning, and more. Furthermore, we only obtained data on percentile rankings for each respondent (not raw score) which vary from year to year over administration periods and are compared to scores of peers in the same level of training across the country. A future consideration would be to utilize raw scores of in-training exams or, more broadly, pass rates on the ABEM qualifying exam, as the latter is already used in program evaluation. This would likely require a much larger sample size to yield statistically significant differences across exam administration periods.

Another potential limiting factor to the generalizability of our results is the intensive time requirement on the part of faculty to set up and conduct the game throughout the year. We estimate that a minimum of 30–50 faculty hours were volunteered throughout the game-play period to tabulate team scores, update leaderboards, and award prizes. This was in addition to time spent designing the game and performing the research component. Implementation of a large-scale game, like ours, would likely necessitate program support for protected faculty time and other resources. Despite these limitations, our study can be used as a basis to design future gamified curricular content and set up future prospective studies on its effectiveness.

## Conclusions

We performed a prospective, pre- and post-intervention survey study to explore the perspective of emergency medicine residents in a newly implemented gamified graduate medical education curriculum. Our results supported the hypothesis that residents reported increased levels of motivation, engagement, and challenge with gamification compared to traditional didactics. Furthermore, residents overwhelmingly indicated agreement with positively worded statements about the impact of gamification on their educational experience. The study produced insufficient evidence to determine whether in-training exam scores are influenced by exposure to gamification compared to a traditional didactic curriculum.

Our study was a first-of-its-kind look into a longitudinal game in an emergency medicine residency program. Although our results are encouraging, medical educators need further research to determine if this increase in motivation, engagement, and challenge will be associated with an increase in examination scores or, more importantly, healthcare outcomes. Theory-based, broader-scale, and prospective studies are needed to further explore and help establish these associations and outcomes.

## Supplementary Information

**Additional file 1: Appendix A.** Game Design.

**Additional file 2: Appendix B.** Play Mechanics and Scoring.

**Additional file 3: Appendix C.** Pre-Intervention Survey.

**Additional file 4: Appendix D.** Post-Intervention Survey.

## Data Availability

Data sharing not applicable to this article as no datasets were generated or analyzed during the current study.
