# Supplementary material for: Gamification of graduate medical education in an emergency medicine residency program
Source: Int J Emerg Med. 2022 Aug 30;15:41. doi: 10.1186/s12245-022-00445-1 (PMC9425934; doi:10.1186/s12245-022-00445-1)

**Appendix A**

**Game Design**

A Game for the AdventHealth East Orlando

Emergency Medicine Residency Program

We created 3 “Teams” consisting of two first-year residents (PGY-1), two second-year residents (PGY-2), and two third-year residents (PGY-3) for a total of six members for each team

Residents earned points for their Individual Score through various tasks, quizzes, and competitions throughout the year. Individual Scores were combined together to get a Total Team Score which was updated (along with an individual leaderboard) at least monthly throughout the year.


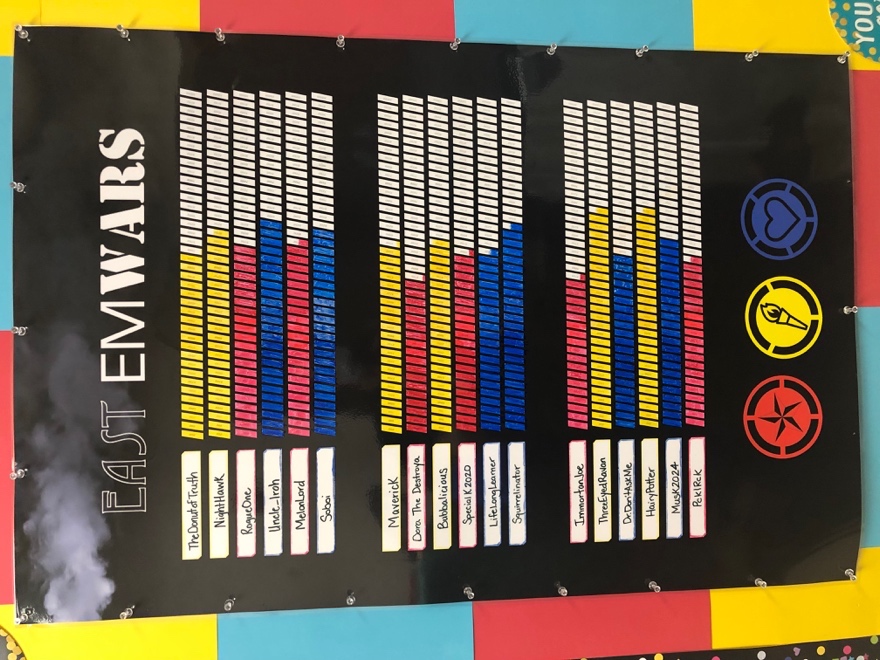

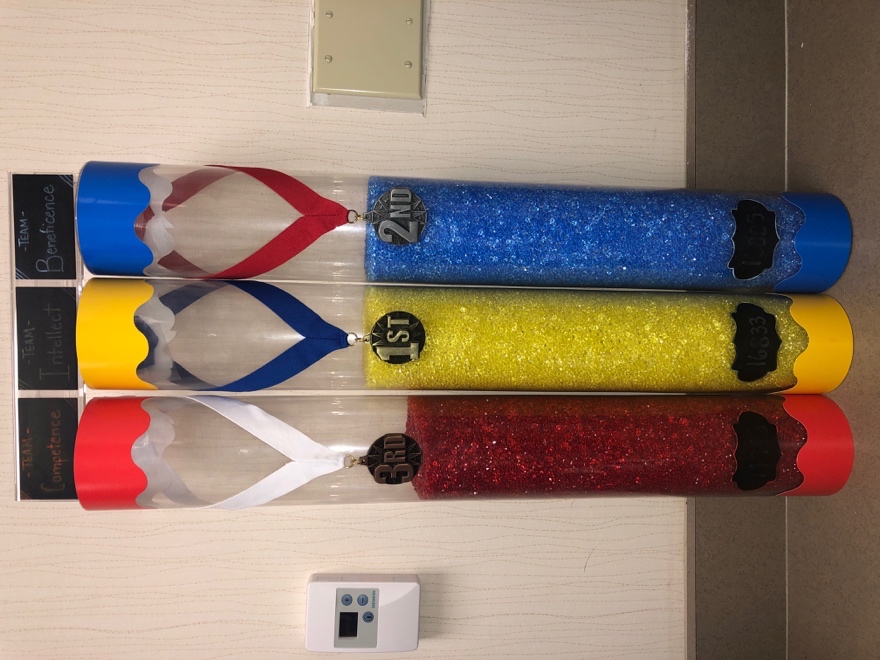


Residents “Leveled-Up” at pre-determined intervals throughout the game and awarded prizes commiserate with their level of achievement (Level 2 Prize = Team T-Shirt, Level 3 Prize = Starbucks GiftCard, Level 5 Prize = Amazon GiftCard, etc.). An Award Ceremony was held at the end of the Game to recognize Team and Individual Winners.


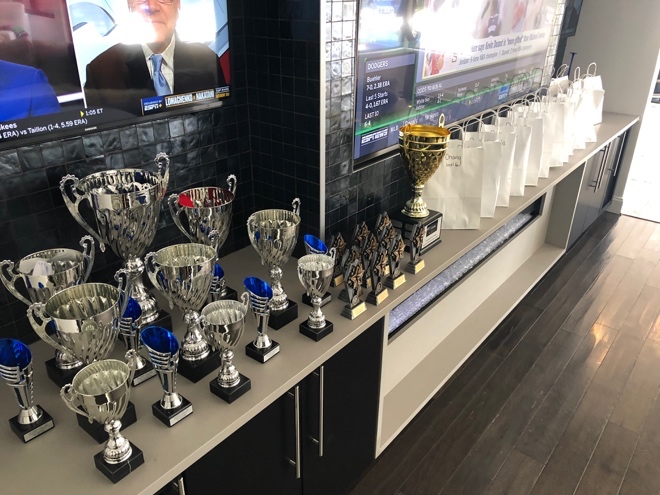

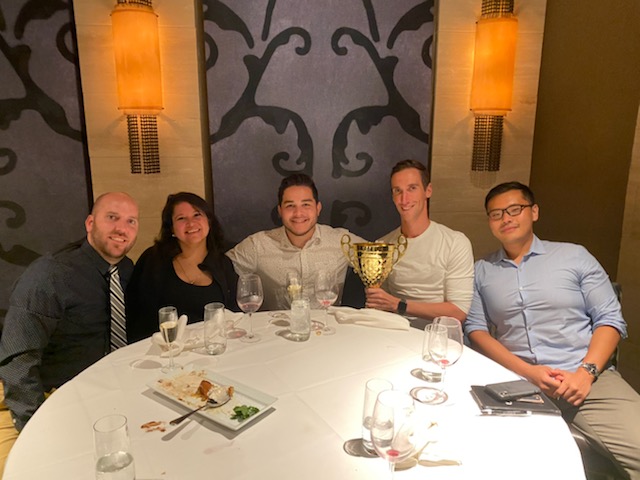

Supplement: Supplementary file 1 — Additional file 1: Appendix A. Game Design. [file 12245_2022_445_MOESM1_ESM.docx]
