# Supplementary material for: Gamification of graduate medical education in an emergency medicine residency program
Source: Int J Emerg Med. 2022 Aug 30;15:41. doi: 10.1186/s12245-022-00445-1 (PMC9425934; doi:10.1186/s12245-022-00445-1)
Supplement: Supplementary file 2 — Additional file 2: Appendix B. Play Mechanics and Scoring. [file 12245_2022_445_MOESM2_ESM.docx]

**Appendix B**

**Play Mechanics and Scoring**

Points were allocated in the following categories:

*#1 – Weekly Quizzes*

Residents in our program are assigned RoshReview quizzes for each instructional content block. Residents were awarded points for timely completion and for each correct answer for a total of 1250 points throughout the entire game.

*#2 – Weekly Assignments*

Residents in our program are assigned one-hour of individualized interactive instruction per week to align with ACGME guidelines allowing the replacement of 20% of in-person conference time with asynchronous learning. Residents were awarded points for timely completion for a total of 1250 points throughout the entire game.

*#3 – Block Challenges*

For this game, we designed “challenges” to align with the objectives for each 4-week rotation block. For example, a resident completing a rotation block in OB/GYN would be awarded points for performing (and appropriately documenting) 10 deliveries. Residents on EM rotation blocks would be awarded points for variety and number of patients evaluated during the block. Residents could potentially earn a total of 2500 points throughout the entire game.

*#4 – Procedure Logger*

Residents in our program utilize New Innovations to log procedures required by the ACGME for successful completion of Emergency Medicine residency training. Residents were awarded 4 points for each approved documented procedure for a max total of 2000 points throughout the entire game.

*#5 – Jeopardy*

Residents in our program competed in their designated Teams in a “Jeopardy”-style quiz game for content review six times throughout the Game (year). Residents were awarded points for toward their individual and team totals based on their ranking with a max total of 1800 points throughout the entire game.

*#6 – Miscellaneous*

We built-in a “Miscellaneous” section to encompass activities that were not planned at the time of the game start. We had anticipated conducting a wilderness medicine race competition and well as an point-of-care ultrasound competition, which were unfortunately not seen to fruition as a result of the COVID-19 pandemic. There were a total of 1200 points allotted for this category, but none were awarded throughout the game.

*Overall*

There were a maximum of 10,000 points available for an individual player to earn (60,000 points per Team). The highest scoring individual finished the game with a total score of 6,674 points; the lowest scoring individual finished the game with a total score of 4,546. The first and second-place teams were separated by a total margin of 35,760 to 35,259. There was anecdotal evidence that this narrow margin was an additional motivator to invested players throughout the entire game, and especially during the weeks leading up to the game’s end.

*Pearls and Pitfalls*

We (game designers) identified three items on our “Pearls and Pitfalls” list prudent to any educational game developer: avoid adding to resident workload, define clear objectives, and cautiously use external motivators (e.g. awards, prizes). First, we made a distinct and purposeful decision to avoid adding anything to resident workload. As you can see above, almost all points were earned by completing tasks already expected from residents in our program. The game created an avenue to provide positive reinforcement for expected behaviors rather than unopposed consequences for shortfalls (as had been the case prior to implementation of the Game). Second, we defined clear objectives and rules for the game before beginning. Every participant was briefed on how points were earned prior to the start, and each player was updated with their Individual and Team Scores on a regular basis. There were large leaderboards displayed in the resident room to allow for easy comparisons throughout the game. Lastly, we were very cautious in the use of extrinsic motivators. Although we did offer small prizes for “Leveling Up” throughout the game and for the winners in the end, all participants were blinded to what those prizes would be from the beginning. We wanted to reward positive participation while acknowledging the known principle that intrinsic, or autonomous, motivation is superior. We highly recommend that future educational game developers regard these “Pearls and Pitfalls” when creating a new game.
