# Supplementary material for: Gamification of graduate medical education in an emergency medicine residency program
Source: Int J Emerg Med. 2022 Aug 30;15:41. doi: 10.1186/s12245-022-00445-1 (PMC9425934; doi:10.1186/s12245-022-00445-1)
Supplement: Supplementary file 3 — Additional file 3: Appendix C. Pre-Intervention Survey. [file 12245_2022_445_MOESM3_ESM.docx]

**Appendix C**

**Pre-Intervention Survey**
