# Supplementary material for: Gamification of graduate medical education in an emergency medicine residency program
Source: Int J Emerg Med. 2022 Aug 30;15:41. doi: 10.1186/s12245-022-00445-1 (PMC9425934; doi:10.1186/s12245-022-00445-1)
Supplement: Supplementary file 4 — Additional file 4: Appendix D. Post-Intervention Survey. [file 12245_2022_445_MOESM4_ESM.docx]

**Appendix D**

**Post-Intervention Survey**
